# Self-Assembly of a 1D Hydrogen-Bonded Polymer from a Hexamethyltetraaza Macrocyclic Nickel(II) Complex and Isophthalic Acid

**DOI:** 10.3390/molecules18066608

**Published:** 2013-06-06

**Authors:** In-Taek Lim, Ki-Young Choi

**Affiliations:** Department of Chemistry Education, Kongju National University, Kongju 314-701, Korea; E-Mail: hak124@kongju.ac.kr

**Keywords:** 1D hydrogen-bonded polymer, nickel(II) complex, tetraaza macrocycle, isophthalic acid, 4/6 coordination number set

## Abstract

The compound [Ni(L)(isoph)_2_][Ni(L)]·8H_2_O **(1**; L = *C*-*meso*-5,5,7,12,12,14-hexamethyl-1,4,8,11-tetraazacyclotetradecane; H_2_-isoph = isophthalic acid) has been synthesized and structurally characterized. Complex **1** exhibits a geometrically symmetric core with a {4/6} coordination number set. The coordination environment around the Ni(1) ion is a distorted octahedron, while the geometry around the four-coordinate Ni(2) is depicted as square planar in 1D hydrogen-bonded infinite chain. The compound crystallizes in the triclinic system *P*-1 with *a* = 8.602(2), *b* = 10.684(7), *c* = 16.550(3) Å, α = 91.04(4), β = 94.09(2), γ = 111.09(4)°, *V* = 1413.9(10) Å^3^, *Z* = 1. The cyclic voltammogram of **1** undergoes one-electron wave corresponding to Ni^II^/Ni^I^ process. The electronic spectra, electrochemical and TGA behavior of the complex are significantly affected by the nature of the hexamethyltetraaza macrocycle and the axial isoph^2−^ ligand.

## 1. Introduction

There has been considerable interest in macrocycles with C-alkyl groups on a polyaza macrocyclic ring and their metal complexes because of their structural and chemical properties, which are often quite different from those of the corresponding unalkylated macrocyclic ligands [1,2,3,4,5,6,7,8,9,10,11,12,13,14,15,16,17,18]. The application of polyaza macrocycle precursors in the synthesis of transition metal(II) macrocyclic complexes, stems mainly from their use as models for protein-metal binding sites in biological systems [19], and as selective complexing agents for metal ions [20]. The structures and chemical properties of such complexes are affected by various factors, such as *C*-alkyl groups in the macrocycle and the type and synthetic differences between the axial ligands. For example, the compounds [Ni(cyclam)(NCO)(H_2_O)](ClO_4_) (cyclam = 1,4,8,11-tetraazacyclotetradecane) [8] and [Ni(dttd)(NCO)_2_] (dttd = 3,14-dimethyl-2,6,13,17-tetraazatricyclo[14,4,0^1.18^,0^7.12^]docosane) [9] exhibit a distorted octahedral geometry, in which the nickel(II) ion is coordinated by four secondary amines and two nitrogen atoms of the isocynate ligand. However, the complex [Ni(Me_4_cyclam)(NCO)](ClO_4_) (Me_4_cyclam = 1,4,8,11-tetramethyl-1,4,8,11-tetraazacyclotetradecane) [8] reveals a distorted square pyramidal geometry, which may be due to the steric constraints of the Me_4_cyclam ligand. On the other hand, the octahedral compound [Ni(L)(OCN)_1.5_(ClO_4_)_0.5_] (L = *C*-*meso*-5,5,7,12,12,14-hexamethyl-1,4,8,11-tetraazacyclotetradecane) [8] presents the peculiarity of the cyanato coordination mode instead of the habitual isocyanate mode. In contrast, the compound [{Ni(L1)}2(μ-OCN)_2_](ClO_4_)_2_ (L1 = dl-5,5,7,12,12,14-hexamethyl-1,4,8,11-tetraazacyclotetradecane) [10] shows that the cyanate ligand acts as an end-to-end bridge with nickel(II) ions in an NiN_5_O octahedral environment. The same synthetic strategy with the azido group leads end-to-end coordination, giving a one-dimensional compound [{Ni(L)(μ-N_3_)}_n_][ClO_4_]_n_ [11]. The different molecular topologies in the complexes may be due to the C-alkyl groups into the macrocycle and different coordination modes of the ligands.

**Figure 1:**
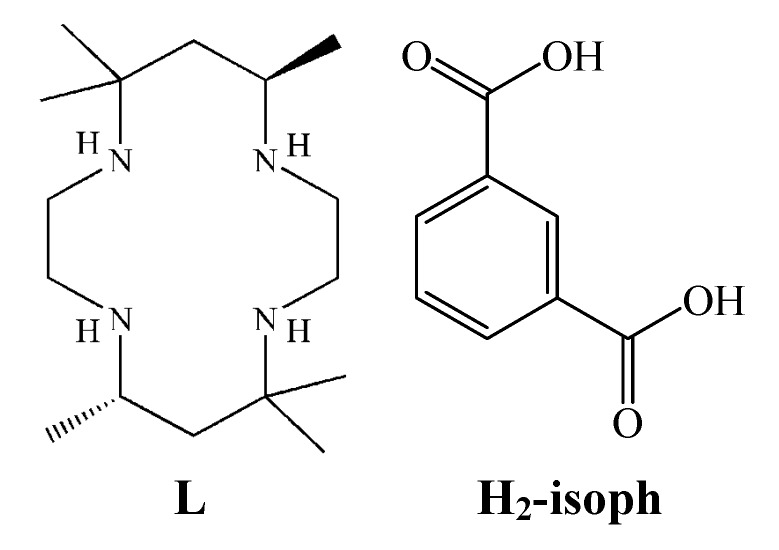
Structure of macrocycle and isophthalate ligand.

In the present work, we report the preparation and characterization of [Ni(L)(isoph)_2_][Ni(L)]·8H_2_O (**1**; L = *C*-*meso* -5,5,7,12,12,14-hexamethyl-1,4,8,11-tetraazacyclotetradecane; H_2_-isoph = isophthalic acid, Figure 1). To evaluate the effect of the hexamethyl tetraaza macrocycle and anionic group, we have investigated the X-ray crystal structure, spectroscopic, and electrochemical properties of the complex.

## 2. Results and Discussion

### 2.1. Description of the Structure

**Figure 2:**
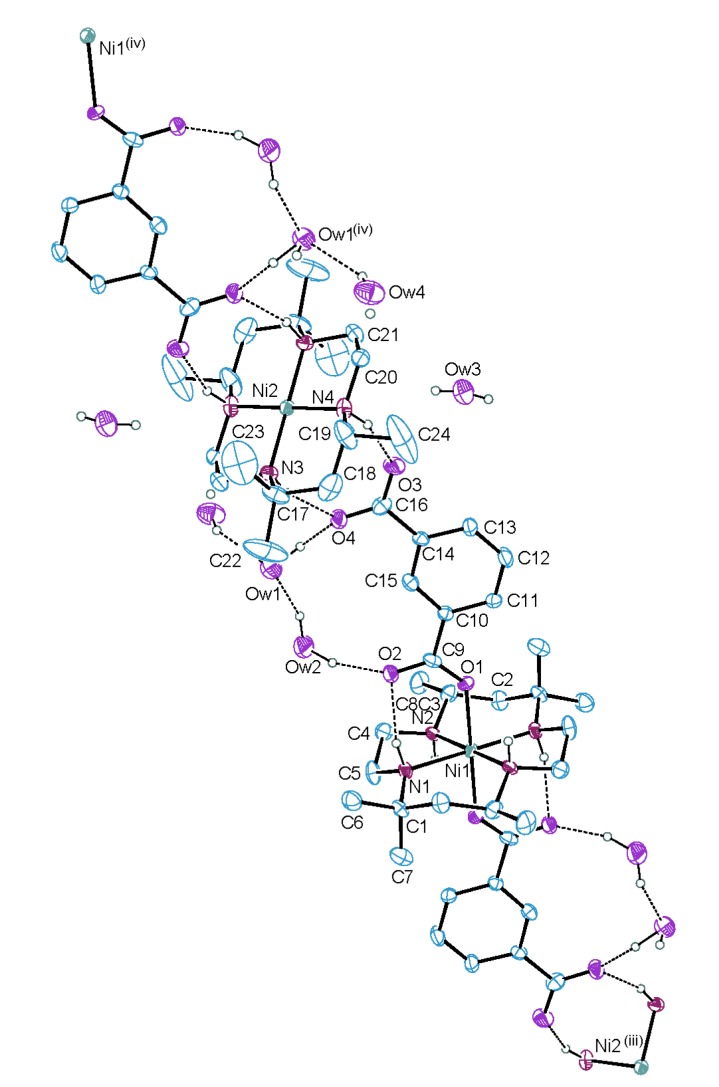
An ORTEP diagram of [Ni(L)(isoph)_2_][Ni(L)]∙8H_2_O (**1**) with the atom-numbering scheme (30% probability ellipsoids shown). The hydrogen atoms other than those participating in hydrogen bonding are omitted for clarity. Symmetry code: (iii) −x, −y+1, −z+1 (iv) −x+1, −y, −z.

**Figure 3:**
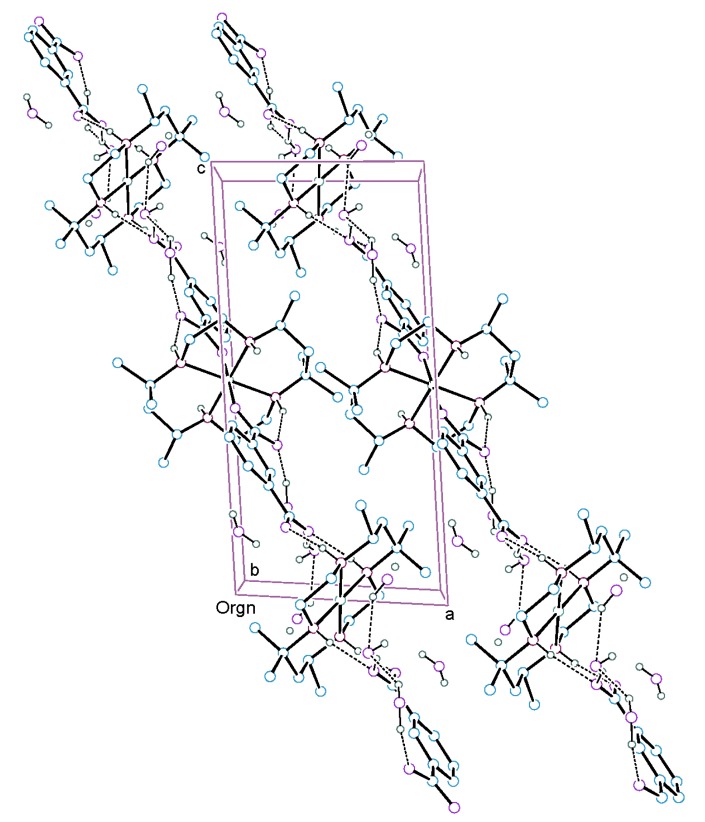
Crystal packing diagram of [Ni(L)(isoph)_2_][Ni(L)]∙8H_2_O (**1**), showing the intermolecular hydrogen bonds as dashed lines. The hydrogen atoms other than those participating in hydrogen bonding are omitted for clarity.

An ORTEP drawing [21] of [Ni(L)(isoph)_2_][Ni(L)]·8H_2_O (**1**) with the atomic numbering scheme is shown in Figure 2. The selected bond lengths and angles are listed in Table 1. The macrocyclic ligand skeleton of the complex takes the most stable *trans*-III(*R*,*R*,*S*,*S*) configuration with two chair six-membered and two gauche five-membered chelate rings. The complex has a geometrically symmetric core with a {4/6} coordination number set. In both structures, the nickel atoms are located at inversion centers. The geometry around the four-coordinate Ni(2) is depicted as square planar with four secondary amines of the macrocycle, while the coordination environment around the Ni(1) ion is a distorted octahedron in which nickel ion is coordinated by four secondary amines of the macrocycle and two carboxylate oxygen atoms of the axial isoph^2−^ ligands. Interestingly, the complex **1** has different topology compared with the compound {[Ni(dttd)(isoph)]·3H_2_O}_n_ [18], which exhibits a 1D coordination polymer with a tetragonally distorted octahedral geometry. This fact may be due to the six methyl groups in the macrocycle, even though the complex **1** has the same axial ligand as the compound {[Ni(dttd)(isoph)]·3H_2_O}_n_. The average Ni(1)-N bond distance of 2.070 Å is typical for those of octahedral nickel(II) complexes with 14-membered tetraaza macrocyclic ligands [6,7,8,9,10]. The Ni(1)-O distance of 2.056(6) Å is shorter than that observed in the related system ({[Ni(dttd)(tp)]·2H_2_O}_n_ [17]: Ni-O = 2.116(3) Å, where tp^2−^ = terephthalate), which has a tetragonally distorted octahedral geometry. The average Ni(2)-N bond distance of 1.896 Å is in the normal range for square-planar nickel(II) complexes with 14-membered tetraaza macrocycles [11,12,13,14,15,16]. The N-Ni-N angles of the six-membered chelate rings are larger than those of the five-membered chelate rings. The intermolecular Ni(1)…Ni(2) distance is 11.641(8) Å, whereas the closest intermolecular Ni(1)…Ni(2)^i^ distance between neighboring strands is 9.607(4) Å. The axial Ni(1)-O(1) bonds are not perpendicular to the NiN_4_ plane as the O_axial_-Ni-N_basal_ angles ranging from 85.6(3) to 94.4(3)°. The dihedral angles (α) between the plane of the carboxylate group and benzene ring involving Ni(1) atom are 2.8(2) and 10.5(9)°, respectively. Moreover, the dihedral angle (β) between the plane of the carboxylate group and NiN_2_O_4_ plane involving Ni(1) atom is 14.6(9)°. The Ni(1)-O(1)-C(9) angle and C(9)-O(1) distance relative to the isophthalate ligand are 133.7(6)° and 1.254(11) Å, respectively. The deprotonated oxygen O(1) among the two isoph^2−^ carboxylic groups is coordinated to the metal center. The secondary amine N(1) of the macrocycle is intramolecular hydrogen bonded to the uncoordinated carboxylic oxygen O(2) of the isoph^2−^ ligand [N(1)-H(1)…O(2)^iii^ 2.804(10) Å, 154.6°; symmetry code (iii) −x, −y+1, −z+1]. Interestingly, the deprotonated oxygen atoms O(3) and O(4) of the isoph^2−^ ligand form intermolecular hydrogen bonds to an adjacent secondary amines N(3) and N(4) of the macrocycle involving Ni(2) atom [N(3)-H(3)…O(4) 2.869(10) Å, 164.0°; N(4)-H(4)…O(3) 2.841(11) Å, 157.8°]. Furthermore, the water molecules also forms intermolecular hydrogen bonds with uncoordinated carboxylate oxygen atoms O(2) and O(4) of the isoph^2−^ ligand [Ow(1)-HO(1)…O(4) 2.676(11)Å, 160(9)°; Ow(2)-HO(3)…O(2) 2.734(12) Å, 163(9)°] and the other water inclusions [Ow(2)-HO(4)…Ow(1) 2.706(14) Å, 147(11)°; Ow(4)-HO(8)…Ow(1)^iv^ 2.829(17) Å, 145(13); symmetry code (iv) −x + 1, −y, −z]. This interaction gives rise to a 1D hydrogen-bonded polymer (Table 2 and Figure 3).

**Table 1:** Selected bond lengths (Å) and angles (°).

Bond lengths and angles			
*Bond lengths*			
Ni(1)-N(1)	2.130(7)	Ni(1)-N(2)	2.009(7)
Ni(1)-O(1)	2.056(6)	Ni(2)-N(3)	1.913(7)
Ni(2)-N(4)	1.878(8)	C(9)-O(1)	1.254(11)
C(9)-O(1)	1.238(11)	C(16)-O(3)	1.196(12)
C(16)-O(4)	1.239(12)	Ni(1)…Ni(2)	11.641(8)
Ni(1)…Ni(2)^i^	9.607(4)		
*Bond angles*			
N(1)-Ni(1)-N(2)	87.0(3)	N(1)-Ni(1)-N(2)^iii^	93.0(3)
N(1)-Ni(1)-O(1)	89.7(3)	N(1)^iii^-Ni(1)-O(1)	90.3(3)
N(2)-Ni(1)-O(1)	85.6(3)	N(2)^iii^-Ni(1)-O(1)	94.4(3)
N(3)-Ni(2)-N(4)	91.3(4)	N(3)-Ni(2)-N(4)^iv^	88.7(4)
Ni(1)-O(1)-C(9)	133.7(6)	O(1)-C(9)-O(2)	127.7(8)
O(3)-C(16)-O(4)	122.6(9)		

Symmetry codes: ^i^ x−1, y, z; ^iii^ −x, −y+1, −z+1; ^iv^ −x+1, −y, −z.

**Table 2:** Hydrogen bonding parameters (Å, °).

D-H^…^A	D-H (Å)	H^…^A (Å)	D^…^A (Å)	D-H^…^A (°)
N(1)-H(1)^…^O(2)^iii^	0.91	1.95	2.804(10)	154.6
N(3)-H(3)^…^O(4)	0.91	1.98	2.869(10)	164.0
N(4)-H(4)^…^O(3)	0.91	1.98	2.841(11)	157.8
Ow(1)-HO(1)^…^O(4)	1.07(10)	1.65(11)	2.676(11)	160(9)
Ow(2)-HO(3)^…^O(2)	1.00(11)	1.76(11)	2.734(12)	163(9)
Ow(2)-HO(4)^…^Ow(1)	0.89(13)	1.92(13)	2.706(14)	147(11)
Ow(4)-HO(8)^…^Ow(1)^iv^	0.82(13)	2.12(14)	2.829(17)	145(13)

Symmetry codes: ^iii^ −x, −y+1, −z+1; ^vi^ −x+1, −y, −z.

### 2.2. Chemical Properties

**Figure 4:**
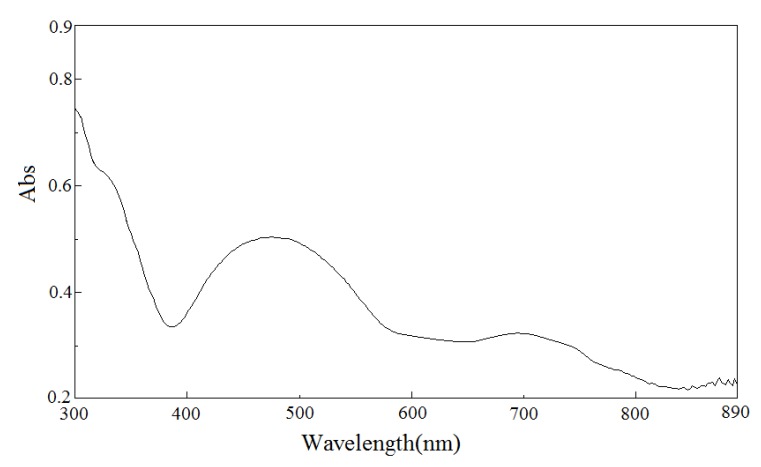
Solid state electronic absorption spectrum of [Ni(L)(isoph)_2_][Ni(L)]∙8H_2_O (**1**) by the diffuse reflectance method at 20.0 ° 0.1 °C.

The IR spectrum of **1** shows a band at 3148 cm^−1^ corresponding to the ν(NH) of the coordinated secondary amines of the macrocycle. Two strong bands exhibit ν_as_(COO) stretching frequency at 1606 cm^−1^ and ν_sym_(COO) at 1357 cm^−1^, respectively. The value of Δν (249 cm^−1^) indicates that the carboxylate groups are coordinated to the nickel(II) ion only as monodentate ligands [22,23]. In addition, a sharp band at 3420 cm^−1^ is associated to the ν(OH) stretching vibration of the water molecule. The UV-Vis spectral data of **1** are listed in Table 3. The solid reflectance spectrum of **1** is also shown in Figure 4. The UV spectrum of **1** in the water solution shows an absorption maximum in the region 260 nm attributed to a ligand-metal charge transfer associated with the nitrogen and oxygen donors [23]. As shown in Figure 4, the solid state electronic spectrum of **1** in the visible region shows three absorption bands at 325(sh), 474, and 696 nm assignable to the ^3^B_1g_→^3^E_g_(T_1g_(P)), ^1^B_2g_→^1^B_1g_, ^3^B_1g_→^3^B_2g_(T_2g_(F)) transitions [3,24]. The transitions at 325 and 696 nm arise from the tetragonal complex while the transition at 474 nm results from the square planar species. However, the visible spectrum of **1** in water solution displays a broad band 458 nm, which has a low-spin d^8^ nickel(II) ion in a square-planar environment [Ni(L)](ClO_4_)_2_ (463nm) [5]. This fact can be understood in terms of the decomposition of the hydrogen bonding or the building block in water solution. The electronic spectrum for **1** clearly support the structure determined by the X-ray diffraction study.

**Table 3:** Electronic spectral data ^a^.

Complex	State	λ_max_/nm (ε/M^−1^ cm^−1^)
[Ni(L)](ClO_4_)_2_^b^	H_2_O	463(80)
	CH_3_CN	469(63)
	Solid	262, 468
[Ni(L)(isoph)_2_][Ni(L)]∙8H_2_O (**1**)	H_2_O	260(5.2 × 10^3^), 458(71)
	Solid	325(sh), 474, 696

^a^ Solution = H_2_O at 20 ± 0.1 °C; Solid = diffuse reflectance; ^b^ Ref. [5].

**Figure 5:**
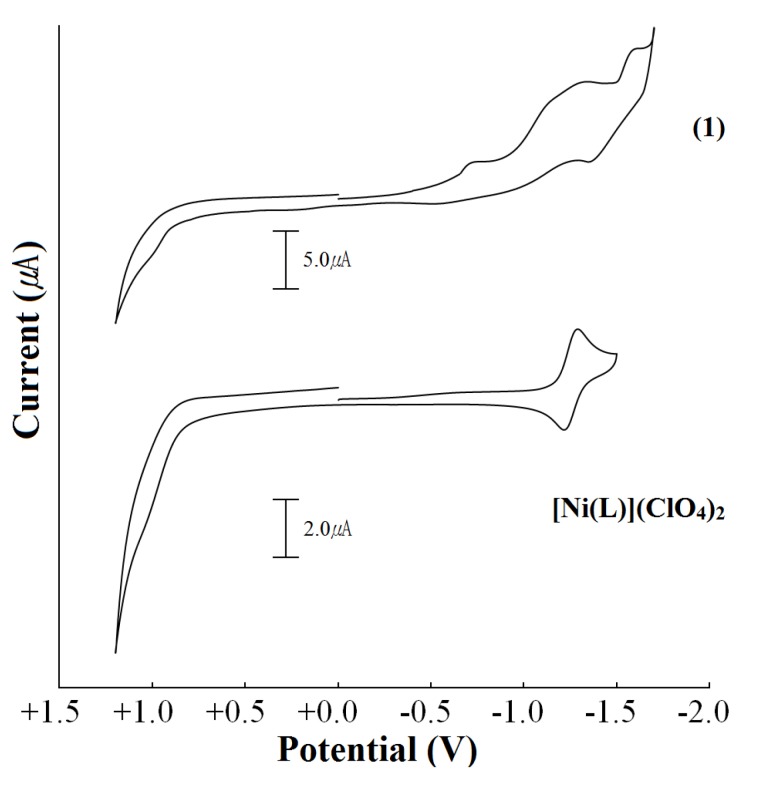
Cyclic voltammograms of [Ni(L)](ClO_4_)_2_ and [Ni(L)(isoph)_2_][Ni(L)]∙8H_2_O (**1**) in 0.1 M TEAP-DMSO solution at 20.0 ± 0.1 °C. The scan rate is 100 mV/s.

Cyclic voltammetric data for **1** in 0.10 M TEAP-DMSO solution are given in Table 4. Cyclic voltammogram of **1** in 0.1 M TEAP-DMSO solution is shown in Figure 5. The H_2_-isoph ligand is electrochemically active in the range of potential studied. Cyclic voltammetry of H_2_-isoph shows two irreversible one-electron reductions at −1.33 and −1.61 V vs. the Ag/AgCl reference electrode. The reduction potential for **1** gives the irreversible one-electron process at −0.76 V vs. the Ag/AgCl refernce electrode, assigned to the Ni^II^/Ni^I^ process. The reduction potentials for **1** are considerably more positive than that for the square-planar [Ni(L)](ClO_4_)_2_ (−1.29V) [5]. This fact may be due to the coordination of the axial isoph^2−^ ligand, which is in agreement with the crystal structure of **1**.

**Table 4:** Cyclic voltametric data^a^.

Complex	Potentials(V) Ag/AgCl
	Ni(II)/Ni(I)
[Ni(L)](ClO_4_)_2_^b,a^	−1.29
[Ni(L)(isoph)_2_][Ni(L)]∙8H_2_O (**1**)	−0.76(i) ^c^

^a^ Measured in 0.10 M TEAP-DMSO solution at 20.0 ± 0.1°C; ^b^ Ref. [5]; ^c^ i = irreversible.

**Figure 6:**
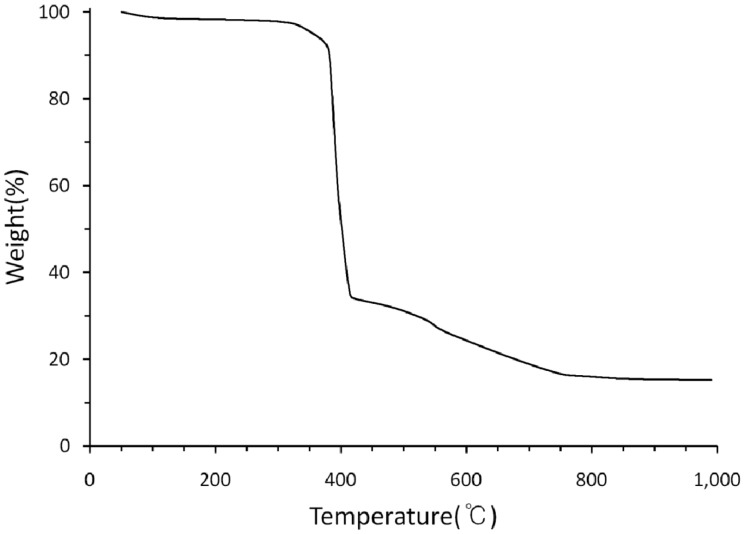
Thermogravimetric curve of [Ni(L)(isoph)_2_][Ni(L)]∙8H_2_O (**1**).

The TGA diagram of **1** is shown in Figure 6. The compound was heated in the temperature range 50–1,000 °C in nitrogen gas. The first weight loss is observed from 30 to 139 °C, which is due to the loss of six water molecules (observed 8.1%, calculated 9.3%). 

A second weight loss corresponding to the macrocycle (observed 51.8%, calculated 49.1%) is found in the temperature range 382–405 °C. On further heating, **1** lost weight between 405 and 756 °C corresponding to two isophthalate ligands (observed 26.7%, calculated 28.3%). Further weigh loss is not observed in the temperature range 756 to 1000 °C corresponding to the greenish black residue of NiO (observed 13.3%, calculated 12.9%).

## 3. Experimental

### 3.1. Materials and Physical Measurements

The chemicals and solvent NiCl_2_·6H_2_O (99.99%), isophthalic acid (99%), and acetonitrile (99.93%) were purchased from Aldrich (Milwaukee, WI, USA) and used without futher purification. The *C-meso*-5,5,7,12,12,14-hexamethyl-1,4,8,11-tetraazacyclotetradecane ligand (**L**) was prepared according to literature method [4,5]. IR spectra were recorded with a Jasco FT/IR-4100 spectrophotometer using KBr pellets. Electrochemical measurements were accomplished with a three electrode potentiostat BAS-100BW system. A 3-mm Pt disk was used as the working electrode. The counter electrode was a coiled Pt wire and an Ag/AgCl electrode was used as a reference electrode. Cyclic voltammetric data were obtained in DMSO solution using 0.10 M tetraethylammonium perchlorate (TEAP) as supporting electrolyte at 20.0 ± 0.1 °C. The solution was degassed with high purity N_2_ prior to carrying out the electrochemical measurements. The DSC and TGA were performed under flowing nitrogen at a heating rate of 10 °C mim^−1^ using a SDT 2960 Thermogravimetric Analyzer. Elemental analyses (C, H, N) were performed on a Perkin-Elmer CHN-2400 analyzer.

### 3.2. Synthesis of [Ni(L)(isoph)_2_][Ni(L)]·8H_2_O *(**1**)*

To a methanol solution of (20 mL) of NiCl_2_·6H_2_O (119 mg, 0.5 mmol) was added **L** (142 mg, 0.5 mmol) and sodium isophthalate (Na_2_isoph) (105 mg, 0.5 mmol) and the mixture was stirred for 1 hr at room temperature. The solution was filtered and left at room temperature until the violet crystals formed. The product was recrystallized from a hot water/acetonitrile (1:1 v/v, 10 mL) mixture. Yield: 56%. Calc. (found) for C_48_H_96_N_8_Ni_2_O_16_: C, 49.76 (49.62); H, 8.35 (8.46); N, 9.67 (9.75)%. IR (KBr, cm^−1^): 3,420 (s), 3269 (m), 3,148 (m), 3,068 (m), 2,962 (m), 1,606 (s), 1,561 (s), 1,460 (w), 1,422 (w), 1,373 (s), 1,357 (s), 1,304 (w), 1,274 (w), 1,186 (m), 1,156 (m), 1,095 (m), 1,073 (m), 1,053 (m), 997 (w), 958 (w), 928 (w), 810 (w), 743 (m), 710 (m), 692 (w), 641 (w).

### 3.3. X-ray Crystallography

Single crystal X-ray diffraction measurement for **1** was carried out on an Enraf-Nonius CAD4 diffractometer using graphite-monochromated Mo-Kα radiation (λ = 0.71073 Å). Intensity data were measured at 293(2) K by ω-2θ technique. Accurate cell parameters and an orientation matrix were determined by the least-squares fit of 25 reflections. The intensity data were corrected for Lorentz and polarization effects. Empirical absorption correction was carried out using ψ-scan [25]. The structure was solved by direct methods and the least-squares refinement of the structure was performed by the SHELXL-97 program [26]. All the non-hydrogen atoms were refinded anisotropically. The hydrogen atoms were placed in calculated positions, allowing them to ride on their parent C and N atoms with *U*_iso_(H) = 1.2*U*_eq_(C or N). A summary of the data collections and details of the structure refinement is given in Table 5.

Crystallographic data for the structural analysis have been deposited with the Cambridge Crystallographic Data Center, CCDC No. 846954 for **1**. Copies of this information may be obtained free of charge from the Director, CCDC, 12 Union Road, Cambridge, CB2, 1EZ, UK (fax: +44-1223-336033; e-mail: deposit@ccdc.cam.uk or http://www.ccdc.cam.ac.uk).

**Table 5:** Crystallographic data.

Data and refinement	
Empirical formula	C_48_H_96_N_8_Ni_2_O_16_
Formula weight	1157.94
Temperature (K)	293(2)
Crystal color/habit	Brown/prism
Crystal system	Triclinic
Space group	*P*-1
Unit cell dimensions	
*a* (Å)	8.602(2)
*b* (Å)	10.684(7)
*c* (Å)	16.550(3)
α (°)	91.04(4)
β (°)	94.09(2)
γ (°)	111.09(4)
*V* (Å^3^)	1413.9(10)
*Z*	1
*D*calc (Mg m^−3^)	1.360
Absorption coefficient (mm^−1^)	0.738
*F*(000)	624
Crystal size (mm^3^)	0.40 × 0.30 × 0.10
θ range (°)	1.23 to 26.37
Limiting indices	−10 ≤ *h* ≤ 10, −13 ≤ *k*≤ 13, −1 ≤ *l*≤ 19
Reflection collected/unique	5729/5137 (*R*_int_ = 0.0817)
Absorption correction	ψ-scan
Max./min. transmission	0.9145 and 0.7122
Data/restraints/parameters	5137/0/369
Goodness of fit on *F*^2^	1.184
Final *R* indices (*I*>2σ(*I*))	*R*_1_^a^ = 0.0811, *wR*_2_^b^ = 0.2224
*R* indices (all data)	*R*_1_ = 0.1742, *wR*_2_ = 0.3391
Weighting scheme	*w* = 1/[σ^2^(*F*_o_^2^) + (0.20000*P*)^2^ +0.0000*P*]
rgest difference peak and hole (eÅ^−3^)	with *P* = (*F*_o_^2^ + 2*F*_c_^2^)/3
	1.731 and −1.199

^a^ *R*_1_ = Σ|| *F*_o_|−| *F*_c_||/Σ| *F*_o_|; ^b^ *wR*_2_ = [Σ[*w*(*F*_o_^2^-*F*_c_^2^)^2^]/Σ[*w*(*F*_o_^2^)^2^]]^1/2^.

## 4. Conclusions 

Complex **1** reveals a geometrically symmetric core with a {4/6} coordination number set. The coordination environment around the Ni(1) ion is a distorted octahedron, while the geometry around the four-coordinate Ni(2) is depicted as square planar in 1D hydrogen-bonded infinite chain. The solid state electronic spectrum of **1** in the visible region shows three absorption bands assignable to the tetragonal and the square planar species. The cyclic voltammogram of **1** displays a one-electron wave corresponding to the Ni^II^/Ni^I^ redox process. The reduction potentials for **1** are considerably more positive than that for the square-planar [Ni(L)](ClO_4_)_2_. The TGA behavior of the complex **1** is significantly affected by the nature of the hexamethyltetraaza macrocycle and the axial isoph^2−^ ligand.

## Supplementary Materials

Supplementary materials can be accessed at: http://www.mdpi.com/1420-3049/18/6/6608/s1.
